# Magnetic field dose effects on different radiation beam geometries for hypofractionated partial breast irradiation

**DOI:** 10.1002/acm2.12182

**Published:** 2017-09-13

**Authors:** Anthony Kim, Stephanie Lim‐Reinders, Claire McCann, Syed Bilal Ahmad, Arjun Sahgal, Justin Lee, Brian M. Keller

**Affiliations:** ^1^ Department of Medical Physics Sunnybrook Health Sciences Centre/Odette Cancer Centre Toronto ON Canada; ^2^ Faculty of Medicine Department of Radiation Oncology University of Toronto Toronto ON Canada; ^3^ Department of Radiation Oncology Sunnybrook Health Sciences Centre/Odette Cancer Centre Toronto ON Canada

**Keywords:** interface effects, magnetic dose effects, Monte Carlo, MRI linac, partial breast irradiation

## Abstract

**Purpose:**

Hypofractionated partial breast irradiation (HPBI) involves treatment to the breast tumor using high doses per fraction. Recent advances in MRI‐Linac solutions have potential in being applied to HPBI due to gains in the soft tissue contrast of MRI; however, there are potentially deleterious effects of the magnetic field on the dose distribution. The purpose of this work is to determine the effects of the magnetic field on the dose distribution for HPBI tumors using a tangential beam arrangement (TAN), 5‐beam intensity‐modulated radiation therapy (IMRT), and volumetric modulated arc therapy (VMAT).

**Methods:**

Five patients who have received HPBI were selected with two patients having bilateral disease resulting in a total of two tumors in this study. Six planning configurations were created using a treatment planning system capable of modeling magnetic field dose effects: TAN, IMRT and VMAT beam geometries, each of these optimized with and without a transverse magnetic field of 1.5 T.

**Results:**

The heart and lung doses were not statistically significant when comparing plan configurations. The magnetic field had a demonstrated effect on skin dose: for VMAT plans, the skin (defined to a depth of 3 mm) D1cc was elevated by +11% and the V30 by +146%; for IMRT plans, the skin D1cc was increased by +18% and the V30 by +149%. Increasing the number of beam angles (e.g., going from IMRT to VMAT) with the magnetic field on reduced the skin dose.

**Conclusion:**

The impact of a magnetic field on HPBI dose distributions was analyzed. The heart and lung doses had clinically negligible effects caused by the magnetic field. The magnetic field increases the skin dose; however, this can be mitigated by increasing the number of beam angles.

## 1 INTRODUCTION

Hypofractionated partial breast irradiation (HPBI) has been proposed as an ablative procedure to replace surgical resection^1^, ^2^ and also for the preoperative/postoperative adjuvant settings.^3^, ^4^ As an ablative procedure, HPBI has been proposed to reduce the treatment burden for those patients who cannot tolerate surgery. As an adjuvant treatment, HPBI aims to reduce the long, protracted radiation therapy (RT) schedule for standard breast conservation therapy (BCT) into a shorter, hypofractionated regimen targeted toward the postoperative bed where the cancer most often recurs.^5^, ^6^ The minimum treatment burden for low‐risk breast cancer patients is the hypofractionated approach—as similar strategies are employed regularly and successfully for the brain and lung tumor sites, this is an attractive clinical solution. As data are accumulating for HPBI for these clinical settings, a variety of techniques have been developed to administer HPBI, such as interstitial high‐dose‐rate (HDR) brachytherapy, permanent seed implants,^7^ intraoperative RT (for surgical candidates), and inversely planned external beam RT.^8^

An exciting development in radiation oncology that shows promise for HPBI is the emergence of MRI‐guided external beam solutions.^9^ Several ambitious efforts are in progress to integrate MRI into a real‐time or near‐real‐time solution for tumor targeting during or immediately prior to radiation delivery. These efforts have produced a number of hybrid MRI‐RT solutions such as the Cross Cancer Institute MRI‐Linac prototypes at the University of Alberta,^10^ the Australian MRI‐Linac concept headed by Paul Keall's research group,^11^ the first commercialized Cobalt‐60/MRI solution from the company ViewRay^12^ and the pre‐commercial Elekta (Elekta AB, Stockholm, Sweden) MRI‐Linac.^13^

Our cancer center is preparing to install a clinical prototype of the Elekta MRI‐Linac. We anticipate that image‐guided, hypofractionated partial breast irradiation will be a good candidate for the MRI‐Linac's capabilities and thus have interest in developing treatment processes to that end. MRI can visualize breast tumors better than CT‐based imaging^14^, ^15^ potentially affording a great improvement in daily geographical matching and tumor delineation than with current state‐of‐the‐art cone‐beam CT (CBCT). Also, with exquisite soft tissue contrast, MRI can lend itself to adaptive radiation therapy (ART), where the MRI guidance can be used for online visualization and contouring of the tumor as it changes shape and size throughout treatment—particularly of interest in a highly deformable organ such as the breast. Finally, MRI‐Linac solutions are attractive for HPBI due to the ability for these advanced platforms to cut down on internal motion margins using MLC tracking or exception gating using guidance from real‐time MRI images or navigator signals. The MRI‐Linac has been suggested by many to be ideal for image‐guided ablative radiation therapy, a treatment paradigm that is expected to become increasingly prevalent in radiation oncology.^9^, ^13^, ^16^ For low‐risk breast cancer patients, the aforementioned technological advantages given by an MRI‐Linac may pave the way for enabling high precision ablative techniques to be employed for intact breast tumors.

The Elekta MRI‐Linac consists of a 1.5 T closed bore magnet with a linac rotating circumferentially about the imaging system and delivering beam through the cryostat. Since the magnetic field is always on, the electrons liberated by the x‐ray photons are perturbed by the ever‐present magnetic force. One of the consequences of this is the “electron return effect” (ERE), where electrons liberated at tissue‐air and tissue‐lung interfaces curl back on themselves due to the Lorentz force, depositing larger doses in tissue at these interfaces.^17^ This can potentially lead to unwanted elevated doses at the skin or other high‐to‐low density interfaces.

The objective of this work is to determine the effects of the magnetic field for HPBI treatment geometries using 3 different treatment beam arrangements: 2–3 beam tangential arrangement (TAN), 5‐beam intensity‐modulated RT (IMRT) and volumetric modulated arc therapy (VMAT). By determining the magnetic field dose effects with these beam geometries, this work can provide good guidance for beam geometry selection in clinical scenarios.

For these comparisons, treatment plans were generated for all beam geometries and optimized with and without the magnetic field *B*_*0*_ = 1.5 T. Throughout this paper, the following abbreviations will be used: TAN, IMRT and VMAT labels indicate plans with no magnetic field effects; TANB0, IMRTB0 and VMATB0 labels indicate plans using the named beam geometries and with magnetic field effects i.e., *B*_*0*_ = 1.5 T. The plans were created with the same isocoverage of the PTV with the comparisons made via the organs‐at‐risk (OARs), which were the skin, heart, and lungs. The hypothesis was that the skin would be increasingly affected by the ERE with increasing number of beam angles and that the heart and lungs would have both increased maximum doses (due to ERE at high‐to‐low tissue density interfaces) and mean doses (due to more low dose wash).

## 2 MATERIALS AND METHODS

### 2.A Patient selection

This study consists of patients from our hypofractionated partial breast irradiation program who are approved for retrospective analysis by our institutional research ethics board. These are breast cancer patients who did not undergo surgery due to metastatic disease or severe medical comorbidities, the intent being local control and reducing symptom burden. Patients with tumors close to the skin were preferentially selected. Five patients were selected for this study. Two of the patients had bilateral disease, but for this study both tumors were considered separately. Table 1 shows the laterality and PTV volumes for all tumors.

**Table 1:** Laterality and PTV volumes listed for all patient tumors

Tumor	Laterality	PTV vol (cc)
1	Left	61.3
2	Left	76.0
3	Left	91.4
4	Right	37.3
5	Right	56.9
6	Right	97.2
7	Right	341.1

### 2.B Treatment planning

The treatment planning system (TPS) used in this study was Monaco (v.5.09.07, developed by Elekta)—this is the TPS that will be used with the Elekta MRI‐Linac. Monaco uses a Monte Carlo radiation transport method accelerated by a fast graphics processing unit currently under evaluation for clinical deployment, called the Graphics Processing Unit Monte Carlo Dose algorithm (GPUMCD).^18^, ^19^ This Monte Carlo dose calculation algorithm can account for the magnetic field effects on the dose deposition by the radiation beam, which for the MRI‐Linac has an energy of 7 MV. We used Monaco for this study as it is possible to simulate and characterize the magnetic field effects using different treatment planning methodologies.

Patients were positioned supine, with the ipsilateral arm raised overhead (for patients with bilateral tumors, both arms were raised overhead). The GTV was contoured on the planning CT with an isotropic margin of 1 cm around the GTV to form the PTV. This PTV margin is likely much larger than required for an MRI‐Linac; however, we used this margin as it was used clinically for our patients. The heart and lungs were previously contoured on the clinically delivered plan. There were two skin contours that were evaluated: Skin3mm and Skin5mm, which were defined as the volumes 3 and 5 mm deep from the patient external contour and encompassing all beam entry and exit points nearby the tumor. Clinically, our cancer center uses the 5 mm depth to define skin contours; however, we included the 3 mm skin depth in order to determine if magnetic dose effects are more prevalent very close to the patient external surface.

Three different treatment beam geometries were optimized/calculated with *B*_0_ = 0 T: 2‐or 3‐beam tangential arrangement (TAN), 5‐beam intensity‐modulated RT (IMRT) and volumetric modulated arc therapy (VMAT). Each of these beam geometries were also optimized/calculated with a transverse magnetic field of *B*_*0*_ **= 1.5 T; recall that in this article these are referred to as the TANB0, IMRTB0 and VMATB0 plans. This results in six different plan configurations per tumor (i.e., 3 beam geometries ×2 states of the magnetic field). Monaco v. 5.09.07 was used in conjunction with the GPUMCD algorithm which uses a Monte Carlo approach for dose calculation. One key distinction of GPUMCD is that the dosimetric effects of the magnetic field are calculated in the plan optimization stage and thus deleterious effects such as the ERE can be partially mitigated by inverse planning.^13^, ^17^

**Figure 1:**
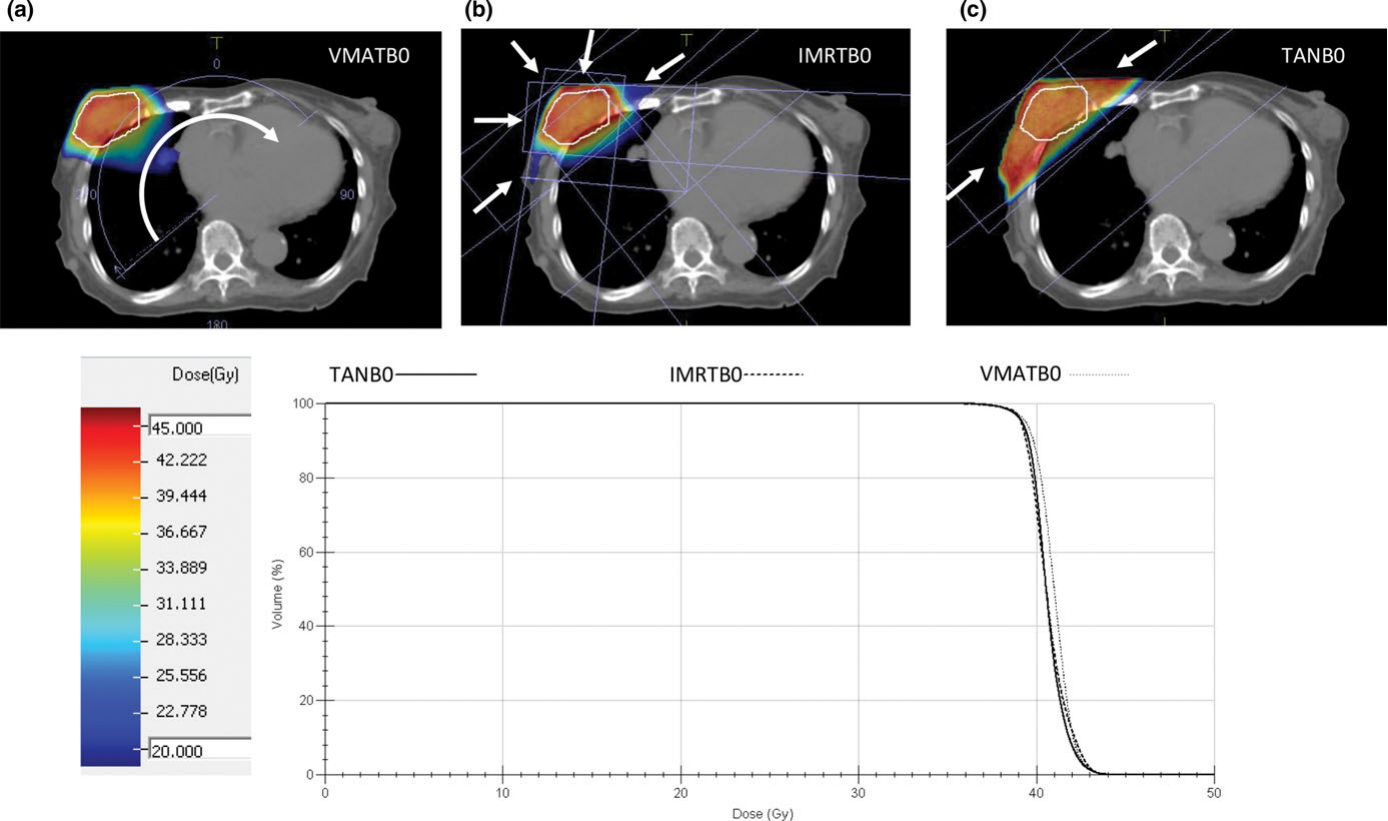
Beam geometries overlaid with screenshots of Tumor 5's tumor anatomy and example dose distributions. VMATB0, IMRTB0, and TANB0 plans (a,b,c, respectively) are shown. The dose**–**volume histogram shows that the PTV coverage of the three plans matches well.

The beam geometries were carefully controlled such that the TAN, IMRT, VMAT, TANB0, IMRTB0, and VMATB0 configurations can be compared. The TAN and TANB0 geometries were 180° parallel opposed (POP) beams arranged to encompass the PTV whilst minimally passing through normal lung tissue. A 3^rd^ beam was added (entering from the anterior‐lateral oblique direction) if it did not pass directly through the heart and was required to produce reasonable coverage—this was the case for Tumors 1, 3, 4, and 6. Clearly the TAN or TANB0 plans will be far less conformal to the target than the equivalent IMRT and VMAT plans; however, this was done because these will be instructive in understanding magnetic dose effects with very low numbers of beam angles. The five IMRT (and IMRTB0) geometry beams were placed within the span of the TAN POP beams at equal gantry spacings 45° apart, with all beams entering from the anterior oblique direction. Since the MRI‐Linac couch cannot rotate relative to the treatment plane, non‐coplanar beams were not considered in this study. The VMAT (and VMATB0) arc was also placed within the span of the TAN POP beams, with the single arcing beam also coming from the anterior oblique direction (see Fig. 1 for an example of all beam geometries with Tumor 5, with *B*_0_ turned on). Each tumor, for all three beam geometries and with *B*_*0*_ on and off, were optimized with identical inverse planning objectives and isocoverage of the PTV (i.e., 99% of the PTV was covered by the 95% prescription isodose). The hot spots within the target were controlled by attempting to keep the V44 < 1%, although breaching this constraint slightly was not a cause for plan rejection (which is also according to our clinical practice). The PTV was evaluated with a modified PTV that retracted from the skin surface by 5 mm. The IMRT objectives strove to minimize dose to the heart and lungs and cover the modified PTV with as homogeneous dose as possible. All cases were planned with a prescription of 40 Gy in five fractions for consistency.

A beam model representing the forthcoming Elekta MRI‐Linac was used for planning all cases. The calculation dose grid size was 1.6 × 1.6 × 1.6 mm and the statistical uncertainty for the Monte Carlo dose calculation was 1% over the entire calculated dose distribution. This was the smallest dose grid size possible for the patient scan size and GPU hardware installed in our Monaco systems. The magnetic field strength (for the *B*_0_‐on plans) was 1.5 T in the transverse direction to match that of the Elekta MRI‐Linac construction. For all plans (*B*_*0*_‐on and *B*_*0*_‐off), a cryostat model was placed in the path of the beam, also to match the Elekta MRI‐Linac to ensure a fair comparison. The only difference between the *B*_*0*_‐on and *B*_*0*_‐off plans was enabling or disabling the 1.5 T magnetic field in the Monaco settings.

The OAR dose‐volume histogram (DVH) values that were collected were: heart max, mean and V32; lung max, mean, V20 and V12.5; Skin3mm max and D1cc; and Skin5mm max and D1cc.

## 3 RESULTS

All plans (for the three beam geometries, *B*_*0*_ on and off, and all tumors) had acceptable PTV coverage with most of the plans (36 out of 42 plans) achieving V44 < 1%.

**Figure 2:**
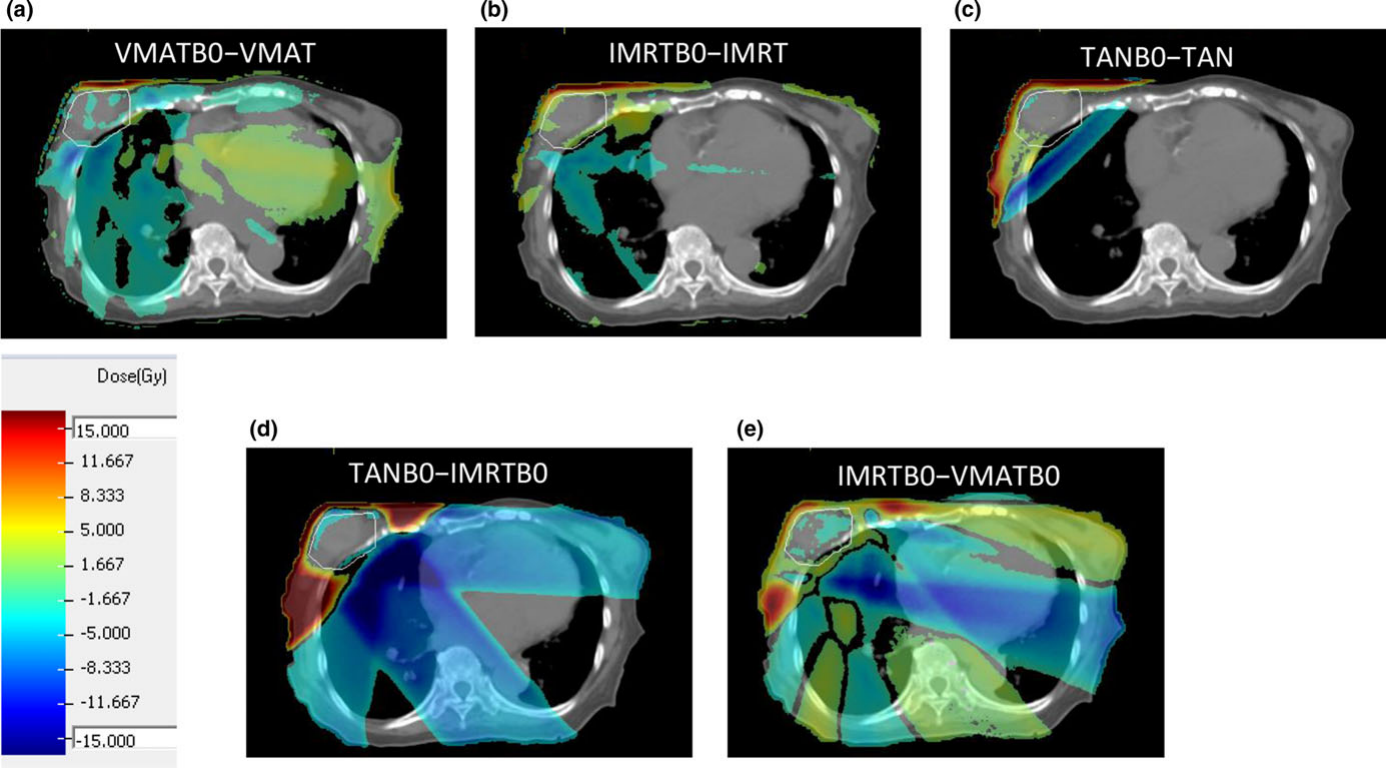
Dose difference maps between plans with different configurations for Tumor 5. (a), (b) and (c) are the B_0_‐on plans subtracted by the B_0_‐off plans for the same beam geometries. (d) and (e) show how, with B_0_‐on, the dose differs with increasing numbers of beam angles. The PTV is outlined in white. Dose differences <±5 Gy are thresholded out of the images for clarity.

Qualitatively, the differences in beam geometries and the dose effects of the magnetic field can be appreciated using dose difference maps. Figures 2(a)–2(c) show dose difference maps that demonstrate the effects of the magnetic field with the same beam geometry (as with Fig. 1, Tumor 5 is shown). All three of these show elevated dose at the very surface of the skin next to and surrounding the tumor. There are also spurious dose differences in the lung‐tissue interfaces and throughout the lung tissue. Figures 2(d) and 2(e) show the effects of fewer beam angles on the dose distribution, particularly at the skin surface. When comparing the TANB0 plan and the IMRTB0 plan, the skin dose is considerably higher for the TANB0 plan. Also, the dose throughout the rest of the patient volume is lower in the TANB0 plan than the IMRTB0 plan (which is clearly expected). When comparing the IMRTB0 plan and the VMATB0 plan, there is similarly a higher dose in the skin for the IMRTB0 plan, with spurious dose differences throughout the rest of the patient. Qualitatively, the magnetic field increases the dose to the skin overlying the tumor; also, the fewer the beam angles, the higher the skin dose.

Table 2 displays DVH parameters averaged over all tumors for each of the plan configurations. In order to parse through the data more efficiently, it was instructive to perform a one‐way analysis of variance (ANOVA) test for a subset of these DVH parameters. Matlab R2015b (Mathworks Inc., Natick, MA, USA) was used to perform these calculations, which are presented in Table 3.

**Table 2:** DVH parameters for all six plan configurations: with B_0_ and without, and for all beam geometries. Data show averages over all tumors (*N* = 7). The ± appendages denote standard deviations across all breast tumors

DVH parameter		VMATB0	VMAT	IMRTB0	IMRT	TANB0	TAN
Heart max dose	(Gy)	22.4 ± 13.1	23.7 ± 12.4	21.4 ± 14.4	22.4 ± 12.2	17.9 ± 20.7	13.9 ± 17.1
Heart mean dose	(Gy)	5.0 ± 2.9	5.1 ± 2.8	4.3 ± 2.4	4.6 ± 2.8	0.9 ± 0.9	0.8 ± 0.9
Heart V32	(%)	0.2 ± 0.6	0.2 ± 0.5	0.2 ± 0.5	0.1 ± 0.3	0.1 ± 0.2	0.1 ± 0.2
Lung mean dose	(Gy)	3.0 ± 2.7	3.4 ± 3.1	2.6 ± 2.1	2.9 ± 2.7	1.3 ± 1.3	1.3 ± 1.2
Lung V20	(%)	2.9 ± 4.4	3.6 ± 5.6	2.0 ± 3.3	2.4 ± 4.8	1.5 ± 3.1	1.4 ± 2.8
Lung V12.5	(%)	5.9 ± 7.7	7.3 ± 9.7	4.4 ± 5.8	5.6 ± 8.5	2.5 ± 3.5	2.6 ± 3.1
Lung max dose	(Gy)	36.6 ± 7.8	33.0 ± 7.4	36.4 ± 8.0	32.6 ± 7.5	34.7 ± 16.3	29.8 ± 11.7
Skin3mm D1cc	(Gy)	36.2 ± 1.5	32.6 ± 2.3	39.6 ± 3.3	33.5 ± 1.6	43.5 ± 5.6	37.1 ± 2.1
Skin3mm max dose	(Gy)	40.9 ± 2.1	39.1 ± 1.3	44.6 ± 4.8	39.3 ± 0.8	49.1 ± 7.0	41.2 ± 1.2
Skin3mm V30	(%)	5.8 ± 2.9	2.9 ± 2.4	8.4 ± 4.3	3.7 ± 2.3	18.1 ± 11.4	10.7 ± 6.9
Skin5mm D1cc	(Gy)	39.2 ± 1.3	38.5 ± 1.5	42.1 ± 2.4	37.8 ± 2.0	44.8 ± 4.5	40.6 ± 1.1
Skin5mm max dose	(Gy)	42.4 ± 1.4	41.7 ± 1.2	45.4 ± 4.3	41.3 ± 0.8	49.9 ± 7.2	43.3 ± 1.1
Skin5mm V30	(%)	8.3 ± 3.8	6.3 ± 3.3	10.1 ± 4.8	7.0 ± 3.5	18.9 ± 11.6	14.3 ± 9.0
PTV V44 Gy	(%)	0.2 ± 0.6	0.0 ± 0.0	2.1 ± 5.3	0.0 ± 0.0	2.6 ± 3.7	2.5 ± 5.8

**Table 3:** Hypothesis testing using one‐way ANOVA for various combinations of the six planning configurations in this study, i.e., plans with B_0_ on (VMATB0, IMRTB0, TANB0) and plans with B_0_ off (VMAT, IMRT, TAN). The bolded entries are those with *P *<* *0.10 and thus are considered statistically significant differences between the data sets

*P* values resulting from one‐way ANOVA									
Data set 1 →	VMATB0	IMRTB0	TANB0	VMATB0	IMRTB0	VMATB0	VMAT	IMRT	VMAT
Data set 2 →	VMAT	IMRT	TAN	IMRTB0	TANB0	TANB0	IMRT	TAN	TAN
Heart max dose	0.856	0.883	0.702	0.993	0.919	0.868	0.986	0.510	0.419
Heart mean dose	0.944	0.812	0.828	0.820	**0.029**	**0.008**	0.919	**0.019**	**0.008**
Lung max dose	0.386	0.373	0.528	0.999	0.958	0.947	0.996	0.832	0.788
Lung mean dose	0.834	0.845	0.965	0.934	0.497	0.313	0.931	0.461	0.281
Skin3mm D1cc	**0.006**	**0.001**	**0.015**	0.241	0.165	**0.006**	0.706	**0.010**	**0.002**
Skin3mm V(30 Gy)	**0.068**	**0.025**	0.167	0.777	**0.055**	**0.013**	0.943	**0.021**	**0.010**
Skin5mm D1cc	0.361	**0.003**	**0.032**	0.201	0.229	**0.007**	0.662	**0.008**	**0.051**
Skin5mm V(30 Gy)	0.313	0.183	0.421	0.900	**0.099**	**0.043**	0.979	**0.077**	**0.052**

**Figure 3:**
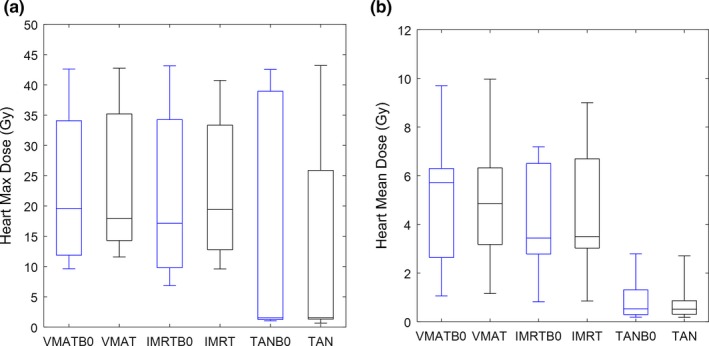
Max heart dose (a) and mean heart dose (b) box‐whisker plots for all six plan configurations. The first and third quartiles are indicated by the ends of the box, with the line in the middle indicating the median. The “whiskers” display the maximum and minimum of the data. No outliers were considered in these plots (these settings apply for all box‐whisker plots).

**Figure 4:**
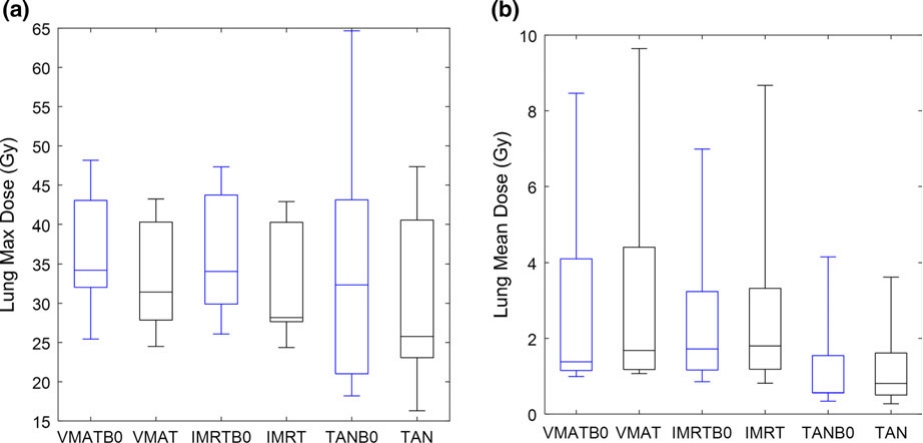
Max lung dose (a) and mean lung dose (b) box‐whisker plots for all six plan configurations.

As expected, the mean heart dose parameters were statistically significant between the TAN datasets and the IMRT & VMAT datasets, regardless of whether the magnetic field was on or off—clearly due to the fact that the TAN and TANB0 beam geometries avoided internal OARs almost completely. The differences between VMAT and IMRT plans, with *B*_*0*_ on or off, are not statistically significant for the heart and lung mean doses. The results of the hypothesis testing for these dose parameters can be visualized more quantitatively in box‐whisker plots shown in Figs. 3(b) and 4(b). The max heart dose generally does not have significant differences across most of the configuration pairings, which can be seen graphically in Fig. 3(a). More interesting is that the differences in the max lung doses have some notable differences when comparing *B*_*0*_‐on and *B*_*0*_‐off for both the VMAT and IMRT geometries in the box‐whisker plot in Fig. 4(a), although they are not strictly statistically significant differences. This is most likely due to the electron return effect on the lung‐tissue interface in the complexly modulated VMATB0 and IMRTB0 plans.

**Figure 5:**
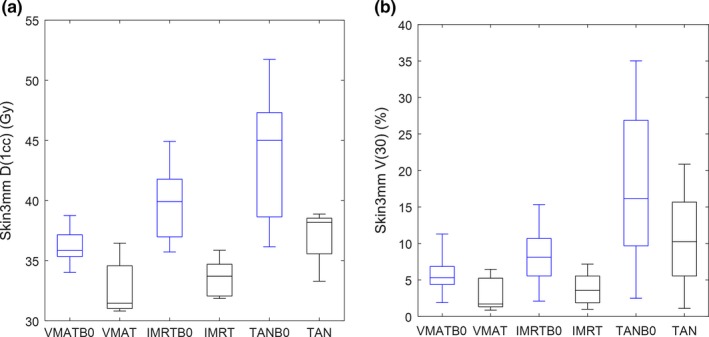
Skin3mm D1cc (a) and Skin3mm V30 (b) box‐whisker plots for all six plan configurations.

**Figure 6:**
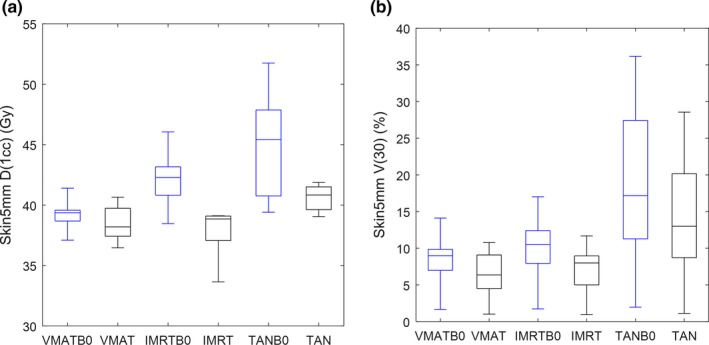
Skin5mm D1cc (a) and Skin5mm V30 (b) box‐whisker plots for all six plan configurations.

The largest difference between plans with different configurations is shown in the skin dose parameters. Many of these differences (between different beam geometries, and between plans with *B*_*0*_ on or off) are statistically significant; moreover, the quantitative differences are readily visualized in the box‐whisker plots in Figs. 5 and 6. In these plots, the D1cc parameter can be seen as a metric for high‐level skin dose, and V30 can be understood as a metric for intermediate dose level. The dose differences between *B*_*0*_ on or off plans are visually larger for the Skin3mm contour than for the Skin5mm contour, which suggests that the main dose effects of the magnetic field are very near the surface.

Table 4 shows the averaged percent differences between plan configurations for the skin metrics shown in the aforementioned box‐whisker plots. The D1cc and V30 increase considerably with *B*_*0*_ turned on, as can be shown in the 1^st^ three columns. The last two columns indicate that the skin dose parameters are reduced considerably with increased beam angles. One last observation from Table 4: our initial observation from the box‐whisker plots that the Skin3mm dose parameters have larger magnitude differences with *B*_*0*_ on or off compared with the Skin5mm dose parameters is verified by the percent difference calculations.

**Table 4:** Averaged percent differences between data sets for selected skin DVH metrics. The formula applied was: %diff = DVHvalue(Data set 1)/DVHvalue(Data set 2)×100% – 100%; this result was averaged over all tumors in the study. The V30 values can be interpreted as the averaged increase or decrease in the volume getting at least 30 Gy

Averaged % differences between Data sets 1 and 2					
Data set 1→	VMATB0	IMRTB0	TANB0	VMATB0	IMRTB0
Data set 2→	VMAT	IMRT	TAN	IMRTB0	TANB0
Skin3mm D1cc	+11%	+18%	+17%	−8%	−8%
Skin3mm V(30Gy)	+146%	+149%	+77%	−28%	−44%
Skin5mm D1cc	+2%	+12%	+10%	−7%	−6%
Skin5mm V(30Gy)	+40%	+53%	+38%	−15%	−37%

## 4 DISCUSSION

This work examined the impact of the magnetic field on OARs for various HPBI beam geometries, namely the TAN, IMRT, and VMAT geometries. For tightly conformal dose distributions, IMRT and VMAT are the likely candidates for HPBI. Although the 2‐ or 3‐beam TAN geometry is less likely to be used in a hypofractionated setting due to reduced conformality, it is instructive to see how very few beam angles affect the OAR doses (particularly that of the skin) in the presence of a magnetic field.

The heart and lung doses were not affected significantly by the magnetic field when using the IMRT or VMAT geometries. The max lung dose (which is a parameter not often looked at as lung is a parallel organ) appears to be slightly increased by the magnetic field for IMRT and VMAT, though in this study was not shown to be strictly a significant difference. The elevated max lung dose in the presence of a magnetic field may be due to the lung‐tissue interface at the chestwall—this is particularly prominent in the TANB0 plans, likely due to the ERE being prominent in treatment plans with very few beam angles. For higher ablative breast tumor doses in an MRI‐Linac, caution is warranted to avoid overdosing the chestwall and risk rib fracture.^20^ It is worth noting that the mean heart doses reported here are higher than will eventually be implemented in an MRI‐Linac because the large PTV margin (1 cm) used in this study will likely be reduced in the future (though this margin reduction is currently unknown for an MRI‐Linac).

Skin dose may very well be an important clinical constraint in dose optimization and prescription of HPBI. One study that demonstrates this is the Canadian RAPID study—trials were stopped due to adverse skin toxicity and poor cosmesis in their accelerated partial breast irradiation study arm with a prescription dose of 38.5 Gy in 10 fractions *b.i.d*.^21^ Strategies to reduce unwanted skin dose for HPBI treated by the MRI‐Linac are thus desirable. For both skin contours (Skin3mm and Skin5mm), there were large differences apparent due to the *B*_*0*_ field. Moreover, the skin doses decreased with increasing number of beam angles, especially with the *B*_*0*_ field turned on (i.e., VMATB0 skin doses were lower than IMRTB0 skin doses, which in turn were lower than the TANB0 plans). These differences are quantified in Table 4. So, one can say that in the presence of a magnetic field, an IMRT plan will have on average an 18% increase in the D1cc dose and a 149% increase in the V30 compared to an IMRT plan with no magnetic field. One strategy to reduce the deleterious effects of the magnetic field could be to use more IMRT beam angles or even a VMAT configuration—if a half arc VMAT geometry is used, Table 4/Column 4 shows that the Skin3mm D1cc and V30 can be reduced by 8% and 28%. Table 4 also demonstrates that the effects of the magnetic field and number of beam angles are more impactful at the shallower depths when comparing the differential values for the Skin3mm and Skin5mm volumes. It is worth noting that the minimum skin thickness definition in the Monaco TPS was limited by the dose voxelation (1.6 mm dose voxels were the smallest that were technically possible with the patients in our study). Creating contours approaching the thickness of a single voxel layer at the surface would have led to confounding partial volume effects, so we decided to set the minimum skin thickness to 3 mm, or approximately twice the voxel size.

Breast tumors are close to the external contour of the patient, resulting in many of the beams entering the patient close to the target and exiting far away from the target (except for tangent geometries). The physical explanation for the observation that skin dose decreases with increasing numbers of beam angles is that the ERE tends to have much less impact at the entry points compared with the beam exit points. Ahmad et al. and Paudel et al. demonstrated this in studies validating the accuracy of Monaco's GPUMCD algorithm with and without the magnetic field, and in heterogeneous phantoms of varying material types and densities.^19^, ^22^, ^23^ With increasing numbers of beam angles (from TAN to IMRT to VMAT geometries) more and more beam angles enter the patient closely to the tumor location. Although all the beams of course exit the patient, only the beam angles near the tumor sum up to a high dose at the patient's skin. Since approximately 50% of the TAN beams at the skin surface are exiting the patient, the ERE has a much larger impact than either the IMRT or VMAT cases where the beam angles are spread far more apart. Another reason for the relation between number of beam angles and skin dose is that opposing beams tend to reduce the ERE effect as demonstrated by many such as the study in Bol et al. analyzing multibeam arrangements for a phantom with a spherical air inclusion.^24^

A similar HPBI study carried out by van Heijst et al. at the University Medical Centre in Utrecht, Netherlands studied the effects of the magnetic field at varying strengths on the OARs for 7‐beam IMRT plans targeting the post‐operative tumor bed.^25^ The authors noted that for this beam arrangement, the skin doses, as quantified by the D2cc, had clinically negligible differences between *B*_0_‐on and *B*_0_‐off. This does not agree with our results for the IMRT geometries; however, the differences in results may be explainable by the fact that our IMRT plans had only five beams and were arranged over 180°. This study shows that the number of beam angles matter, and it is likely that beam arrangement also matters. Another possible reason for the differences between the Utrecht study and this one is that the Utrecht study defined skin as the 5 mm rind below the external surface; the depth of skin seems to matter as suggested by the results in this study, where the impact is seen mainly in the Skin3 mm volume (see Figs. 5 and 6). Another reason for the differences may be because our study uses Monaco, the TPS that will be used for the clinical MRI‐Linac, whereas the van Heijst study uses a specially developed in‐house MRI‐Linac TPS for their work.

It is worth commenting on the relative impact of the electron contamination (EC) compared with the ERE at the surface of the patient nearby the tumor. The Elekta MRI‐Linac beam model used in this study only models the photon fluence incident on the patient. The cryostat is modeled in such a way that photons traversing through it are Compton scattered but do not generate electrons. The reason for this setup is because any electrons generated above the patient will be swept away longitudinally by the transverse magnetic field, so that with *B*_0_ on there will be effectively zero EC. Hence, not including EC is computationally economical. Therefore, in this study, EC is not considered at all at the patient surface in the MC algorithm. This may not be the case if *B*_0_ is turned off in reality. This raises the questions: in this study, was it realistic to neglect EC liberated in the cryostat if the *B*_0_ field was turned off, and is the EC a large contribution to skin dose in a standard linac? To resolve this, we adapted work we performed validating Monaco's GPUMCD algorithm using the Geant4 MC platform.^19^ A model of the MRI‐Linac beam and cryostat were modeled in Geant4 with a 30 × 30 × 30 cm phantom in the path of the beam. The percent depth dose for a 5 × 5 cm field (representative of the field sizes used in this study) was generated with and without the electrons liberated from the cryostat considered in the calculation, with the *B*_0_ field turned off. The EC liberated from the cryostat only accounted for 0.8% difference at the surface of the phantom (as normalized to *d*_max_). Zhu et al. demonstrated that the EC contribution is similarly this low in their Monte Carlo examination of an 8 MV beam from a standard linac.^26^ This calculation and the literature demonstrates that it was reasonable to neglect the EC contribution for this study—by far, the dominant surface effects at the field sizes we were using comes from photon backscatter and (with *B*_0_ on) the ERE.

Often, the magnetic field ERE is a deleterious effect for low numbers of beam angles due to elevated surface dose; however, in some scenarios the ERE may be clinically desirable. This leads to the idea of “magnetic bolusing”, i.e., using fewer beam angles in an MRI‐Linac strategically placed and optimized in such a way to deliberately elevate the surface dose in the presence of cutaneous disease. As can be seen in Table 4, the magnetic field results in considerable increases in the Skin3mm D1cc (+18%) and Skin5mm D1cc (+12%) for the five beam IMRT breast plan. For breast cancer, lymphovascular invasion or tumor eruption through the skin are potential indications for bolusing the patient skin to elevate the surface dose. For Head & Neck cancer, often disease extends to the surface requiring bolus to elevate the skin dose. Magnetic bolusing—if possible—would have the obvious benefit of eliminating (or at least reducing the thickness of) physical bolus. This would be advantageous in the circumstance where a physical bolus may not fit the original simulation anatomy due to patient tumor shrinkage or growth. We suggest that magnetic bolusing is an area to explore, and propose two questions: (a) Can magnetic bolusing be used for a clinical advantage? and (b) How many beams, what beam arrangement, and what optimization strategy can be used to cause a magnetic bolusing effect?

To our knowledge, this is the first study to compare HPBI plans with different beam geometries and also compare plans with the 1.5 T magnetic field turned on and off. In particular, the comparison between IMRT and VMAT and the magnetic field dose effects on both geometries is novel in the context of HPBI. The work by van Heijst et al. from Utrecht examined magnetic field effects on 7‐beam IMRT plans for HPBI; this present study further explores these themes by observing that skin dose is significantly impacted not only by the magnetic field but also varies with depth and varies when increasing the number of beam angles such as with a VMAT implementation.

## 5 CONCLUSION

The impact of a 1.5 T magnetic field transversely placed to the radiation beam on HPBI dose distributions was analyzed using TAN, IMRT and VMAT beam geometries. The heart and lung doses are minimally impacted by the presence of the magnetic field, with the exception of the max lung dose which may be attributed to the ERE. The magnetic field increases the skin dose; however, the skin dose decreases with increasing number of beam angles. The data suggest that the effects of the ERE and the beam geometry are more impactful on skin dose if evaluated at shallower depths, as we analyzed the skin dose from two different depths: 0–3 mm and 0–5 mm. We expect that there would be lower ERE impact on plans where the majority of the beam angles causes the beam to enter into and nearby the tumor, since ERE is maximized at the exit point.

## CONFLICTS OF INTEREST

The treatment planning system was provided by Elekta AB, Stockholm, Sweden.
